# Tree species matter for forest microclimate regulation during the drought year 2018: disentangling environmental drivers and biotic drivers

**DOI:** 10.1038/s41598-022-22582-6

**Published:** 2022-10-20

**Authors:** Ronny Richter, Helen Ballasus, Rolf A. Engelmann, Christoph Zielhofer, Anvar Sanaei, Christian Wirth

**Affiliations:** 1 Systematic Botany and Functional Biodiversity, Institute for Biology, Leipzig University, Johannisallee 21, 04103 Leipzig, Germany; 2 German Centre for Integrative Biodiversity Research (iDiv) Halle-Jena-Leipzig, Puschstraße 4, 04103 Leipzig, Germany; 3 Geoinformatics and Remote Sensing, Institute for Geography, Leipzig University, Johannisallee 19a, 04103 Leipzig, Germany; 4 Chair of Physical Geography, Institute for Geography, Leipzig University, Johannisallee 19a, 04103 Leipzig, Germany; 5 Max-Planck Institute for Biogeochemistry, 07745 Jena, Germany

**Keywords:** Climate-change ecology, Forest ecology, Climate sciences

## Abstract

Tree canopies are considered to effectively buffer climate extremes and to mitigate climate change effects. Droughts, which are predicted to become more frequent in the course of climate change, might alter the microclimatic cooling potential of trees. However, our understanding of how microclimate at the tree canopy level is modulated by environmental and tree characteristics and their interactions is still limited. Here, we investigated canopy temperature regulation for five mature co-occurring tree species for two contrasting hydrological situations during the severe drought in 2018. Even though we observed a significant drought-induced decline in canopy cover and transpiration across tree species, we found evidence that differences in the water use strategies of trees affected cooling mechanisms differently. Although a large share of the variations in the cooling potential of trees was explained by direct and indirect effects of meteorological factors, we identified a gradual shift in importance from latent heat flux to components defining the magnitude of sensible heat flux on the energy budget of tree as the drought gained severity. The decrease in latent heat fluxes, approximated by sap flow rates, furthermore resulted in a reduced cooling potential and an equalization of tree species canopy temperatures.

## Introduction

Forest ecosystems have been shown to effectively buffer climate extremes and mitigate climate change effects below the canopy^1–5^ as they create a distinct microclimate, which is decoupled from their surrounding with substantial deviations between ambient air temperature and temperatures within and below the canopy^3,4,6–9^. Globally, understory temperatures in forests were found to be on average 1.7 ± 0.3 °C (mean) and 4.1 ± 0.5 °C (maximum), cooler than ambient temperatures^4^, which is of greater magnitude than the global warming of land and ocean temperatures (0.99 °C) over the past century^10^. However, the magnitude of this cooling effect differs between forest stands. Forest composition appears to play a major role for cooling^8,11^ as tree species differ substantially in morphological and physiological characteristics influencing the energy balance^12^. The tree species influence is expected to be highest in the canopy and in mixed stands, which may result in a three-dimensional mosaic of distinct microclimates^9,13^. These create ecological thermal niches and may thus influence diversity and composition of floristic and faunistic communities^5,14–17^. This complex ‘heatscape’ also feeds back on the physiological performance of trees themselves^18,19^, and thus directly or indirectly affects tree health^20^, ecosystem functioning^20,21^ and therefore also ecosystem services provided by forest ecosystems^22,23^. The capability of forests to maintain microclimate regulation is thus of increased importance when being faced climate change^4^.

In general, trees cool their canopy microclimate by regulating the local energy budget via the interplay of two contrasting mechanisms, namely by evapotranspiration, where water is converted to water vapor by extracting energy from the local environment^24^, and also by absorbing and reflecting the incoming shortwave radiation at the top canopy which prevents the shaded canopy parts below from warming up^6^. While in hot and arid areas the cooling potential of trees can largely be attributed to shading effects^11,25,26^, cooling by transpiration may become an important driver of canopy temperatures in the temperate zone^6,11^. However, more frequent and longer lasting hot and dry periods, recent phenomena related to climate change^10,27–30^, typically cause a reduction in tree transpiration rates^31^. Thus, for the temperate zone, the contribution of the latent heat flux to the local energy budget might be reduced, which in turn would lead to an overall decrease in the cooling potential provided by trees. Davis et al.^3^ even found evidence that some forests will lose their capacity to buffer climate extremes as sites become increasingly water limited.

Differences in the cooling effectiveness of trees species^9,11,32^ were found to be caused by complex interactions of meteorological factors as well as leaf, canopy and hydraulic characteristics of trees^12,33,34^. The amount of incoming solar irradiance being reflected or absorbed by the canopy or transmitted through the canopy, depends on the total leaf surface area available, and thereby determines the degree of canopy warming^35^. Leaf temperature needs to be regulated via transpiration to avoid leaf damage^36^. Tree transpiration is highest at high solar irradiance, high temperatures and consequently high water pressure deficits (VPD), provided that water availability is not limited^37–39^. When being exposed to water stress and high ambient temperatures, trees species as e.g. English oak (*Querucs robur* L.) tend to close their stomata early on to maintain high leaf water potentials, whereas more water spending species as e.g. European ash (*Fraxinus excelsior* L.) continue to transpire to maintain their photosynthetic activity and to provide sufficient leaf cooling^40–42^. Low leaf water potentials and beginning leaf wilting, can cause reversible changes in leaf inclination, towards a more vertical position or even leaf abscission if leaves are irreversible damaged, which leads to higher canopy transparency and in turn reduces the potential of cooling provided by shading^43^. The ability of a tree species to maintain canopy cooling during drought events might thus be strongly related to their water use strategies and their response dynamics which are likely to induce shifts in the species rank order of canopy temperatures as drought stress continues. However, little is known about the species signature and temporal dynamics of the influence of extended droughts on within-canopy temperatures.

As the effects of canopy structure, tree hydraulic characteristics and the environmental template on the energy budget are not uniform throughout the canopy, the canopy temperatures not only differ between tree species, but also vertically in the tree crown^12,44^. Canopy transparency, for instance, which is considered to have the greatest effect in temperature regulation as it affects both shading and transpirational cooling^11,33,36^, exerts opposing effects on temperatures at the upper and lower canopy^44^. While the upper canopy surface temperature was found to increase with canopy density due to an increase in absorption of solar radiation^45^, lower canopy temperatures decrease with increasing canopy density due to stronger radiative shielding^46^. Species-specific vertical gradients of leaf morphology, leaf angle and leaf area density were found to additionally modulate the transmission of radiation through the tree canopies^47,48^. Also, the amount of cooling provided by transpiration varies along a vertical gradient as stomatal density per leaf area and stomatal conductance increases and the distribution of leaf area density varies along the height of a tree^49^. Differences in the magnitude and the form of those gradients affect canopy temperatures within tree crowns, but might also cause temperature differences between species at the same height layer. For instance, Richter et al.^9^ found frequent and pronounced interspecific temperature differences of up to 4 °C during midday for the top and the bottom canopy, but less distinct temperature differences for the middle position of the tree crown. However, due to the restricted access to forest canopies in most studies, the biophysical and meteorological conditions that cause and shape those interspecific temperature variations along the height gradient, as well as their relative importance and their interactions, remain largely unknown.

High temperatures and low soil water availability—as typical for recent hotter-drought events—additionally modify temperature gradients within tree crowns due to their impact on physiological processes and structural tree characteristics. Especially high midday temperatures force a strong stomatal regulation at the top canopy to prevent leaf water loss, which could shift the transpirational cooling to occur at lower canopy layers^19^. Gallego^39^, for example, has shown that the relative contribution of different height layers on total transpiration rates varies with soil moisture availability. Peiffer et al.^50^ demonstrated for beech, a water saving species, that the vertical gradient of stomatal conductance within the crown is strongly reduced under extreme soil water depletion, whereas less reduction in the vertical gradient of stomatal conductance can be expected for water spending tree species. Moreover, drought-related leaf damage or premature senescence can reduce both transpirational cooling and shading^51^. Whether the shift from latent heat fluxes—being under strong species-specific control—to sensible heat fluxes as stomata close during drought conditions results in an equalization of interspecific canopy temperatures is still debated. For example, Richter et al.^9^ found a low interspecific variability when comparing remotely sensed canopy temperatures in broad-leaved tree species during a moderate drought event, whereas McGloin et al.^52^ and Schwaab et al.^53^ found an opposite tendency when comparing broad- and needle-leaved tree species. However, in how far direct and indirect effects of tree species characteristics and the environmental template on interspecific differences in canopy temperatures along the height gradient change as drought conditions become more severe remains currently unresolved.

To predict the consequences of a changing environment on the cooling potential of forest stands, it is therefore of high importance to capture the essential drivers of several processes in the soil-plant-atmosphere continuum that cause and shape the variations in the cooling efficiencies of contrasting tree species^12,44^. In this study, we aim at disentangling direct and indirect effects of tree characteristics and the environmental template, such as incoming solar radiation, wind speed and ambient temperature, on the cooling potential on midday temperatures for two contrasting hydrological situations (‘moist’, ‘dry’) during the year 2018, the most severe and long-lasting summer drought and heat wave ever recorded in Central Europe^54^. We therefore measured air temperature profiles, sap flow and canopy cover of five mature co-occurring broadleaved tree species using a canopy crane facility which is located within a floodplain forest in Leipzig (Saxony, Germany). As both, the meteorological and biophysical impact on the cooling potential changes with increasing distance to the top canopy^12^, we expected those effects to not only vary between tree species and spatially within the tree crown, but also with hydrological situations. We therefore aim at answering the following research questions:

Q1: Do vertical differences in canopy temperature of tree species change for two contrasting hydrological situations and do these potential changes result in a changed species rank order?

Q2: What are the effects of tree species characteristics and the environmental template such as incoming solar radiation, wind speed and ambient temperature on interspecific differences in canopy temperatures along the height gradient?

Q3: Do the effects of tree species characteristics and the environmental template on interspecific differences in canopy temperatures along the height gradient change as drought conditions become more severe and does the decrease in latent heat fluxes, approximated by sap flux rates, result in an equalization of tree species canopy temperatures?

## Results

### Climatic conditions and soil moisture status

**Figure 1:**
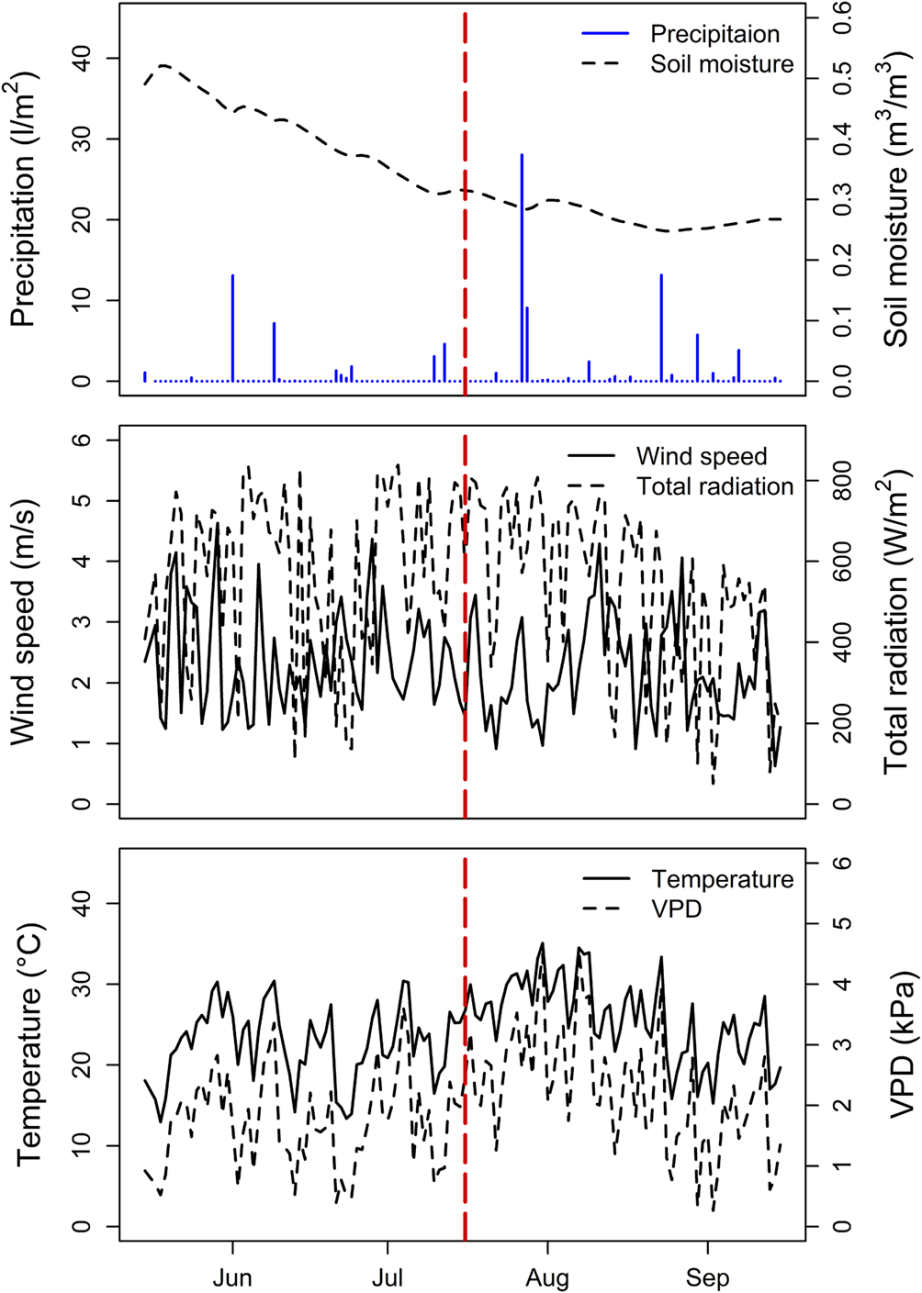
Daily midday (12:00–14:00 CET) average of total radiation, vapor pressure deficit (VPD), wind speed and soil moisture, as well as, total daily precipitation between May 15th and September 15th, 2018, dotted red line separates ‘moist’ and ‘dry’ period.

With a total precipitation of 109 l/m^2^ the investigation period between May 15th and September 15th in 2018 was remarkably dry. In total, we recorded only 14 days with daily sums of precipitation > 1 l/m^2^. Nevertheless, the persistent drought could initially be buffered to some extent by the soil water. This resulted in a constant step decrease of the initially high volumetric soil water content (~ 0.5 m^3^/m^3^) until a local limit of 0.31 m^3^/m^3^ was reached on July the 10th. From this point onwards, soil moisture remained at a low level, which reached its absolute minimum (0.25 m^3^/m^3^) close to the permanent wilting point of vegetation reported for clay soil^55^ on August 24th (Fig. 1).

Based on these observations, the investigation period was classified into a ‘moist’ (61 days) and a ‘dry’ period (62 days), with the dry period starting on July the 16th as we intended to generate a balanced dataset with an equal number of observations for each period (‘moist’, ‘dry’). Based on this classification, soil moisture was found to be lower (by 0.13 m^3^/m^3^) for the dry period (*p* < 0.001). The mean midday ambient temperature during the study period was 24.2 °C. It was significantly higher (by 3.14 °C, *p* < 0.001) during the dry period. Thus, also the vapor pressure deficit VPD (mean at 1.99 kPa), which is highly correlated (r = 0.95, *p* < 0.001) to ambient temperature, was found to be higher (0.55 kPa) during the dry period (*p* < 0.001). However, radiation, windspeed and precipitation were not found to be significantly different between both periods.

### Morphological and physiological tree characteristics

**Figure 2:**
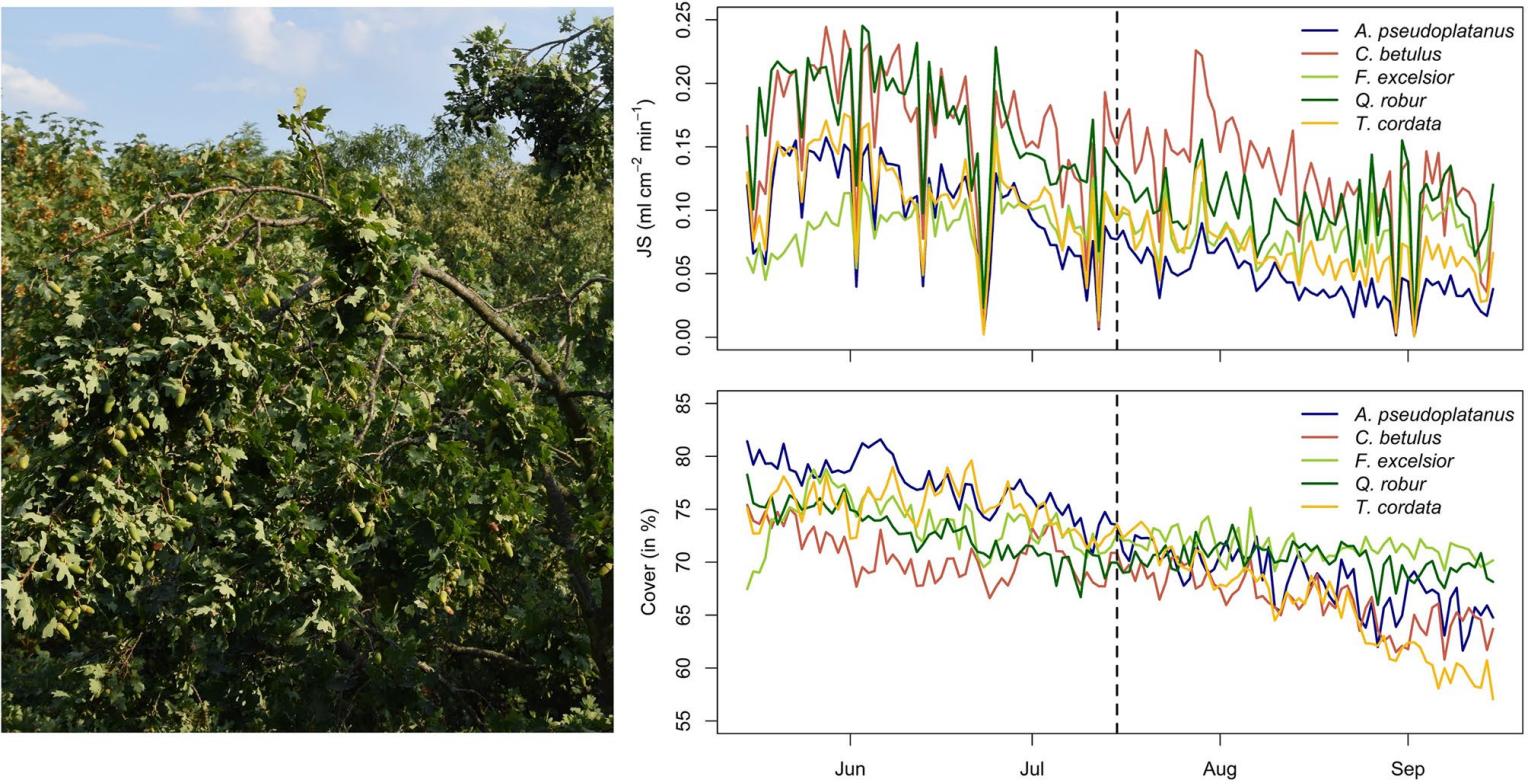
Overhanging branches of *Q. robur* in the top canopy layer during the dry period (left). Species comparison of average daily midday (12:00–14:00 CET) sap flow density (JS) and canopy cover between May 15th and September 15th, 2018, dotted line separates moist and dry period (right).

In parallel with the loss in soil moisture, we found for all tree species tested, a continuous decrease in sap flow and canopy cover over the investigation period (Fig. 2). When comparing species-specific sap flow characteristic for the moist and the dry period (Table 1, Supplementary Tables S7, S8 online), the highest reduction of sap flow was observed for the water saving species *Q. robur* (0.069 ml cm^−2^ min^−1^) and *A.* *pseudoplatanus* (0.058 ml cm^−2^ min^−1^), whereas the water spending species *F. excelsior* showed only a weak albeit significant reduction in transpiration (0.009 ml cm^−2^ min^−1^). Notable is the fast recovery in transpiration in the end of July, which was most pronounced for *C.* *betulus,* and might be related to the concurrent precipitation event during this period, even though this event did not result in a remarkable increase in soil moisture (Fig. 1). The shortage of water heavily affected the canopy structure (Fig. 2). From mid-July onwards, we observed for *Q. robur* trees, due to the loss in turgor pressure, a pronounced drooping of leaves and even branches of up to 5 cm in diameter (Fig. 2, left). The canopy cover in tree species with dense canopies such as *A. pseuoplatanus* and *T. cordata* decreased to levels of canopy cover typical for tree species with transparent canopies such as *F. excelsior* and *Q. robur*. Irrespective of the hydrological situation, an unusually low canopy cover was observed for *C. betulus*. When being compared to moist conditions, the highest decrease in canopy cover was observed for *T. cordata* (by 9.34%) and *A. pseudoplatanus* (by 9.04%), while lowest reduction in canopy cover was observed for *F. excelsior* (by 2.21%).

**Table 1:** Model results when testing for the change in mean centered sap flow density (JS) and canopy cover (%) for dry conditions being compared to moist conditions.

	Sap flow		Cover	
Species	Estimate	*p*-value	Estimate	*p*-value
*A. pseudoplatanus*	− 0.058	< 0.001	− 9.04	< 0.001
*C. betulus*	− 0.038	< 0.001	− 4.37	< 0.001
*F. excelsior*	− 0.009	< 0.001	− 2.21	< 0.001
*Q. robur*	− 0.069	< 0.001	− 2.59	< 0.001
*T. cordata*	− 0.045	< 0.001	− 9.34	< 0.001

### Species-specificity of within canopy temperature profiles

**Figure 3:**
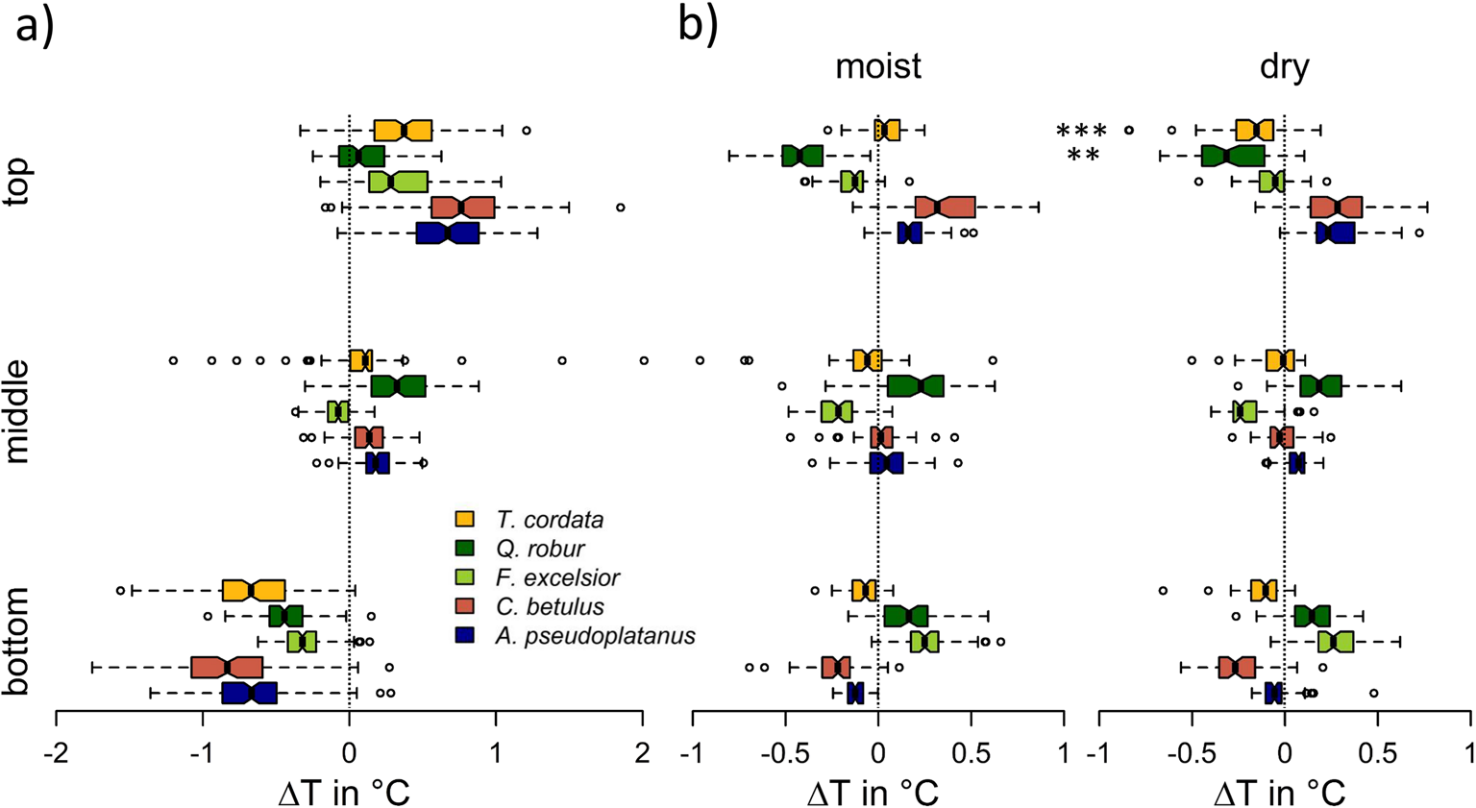
(**a**) Variations in species-specific canopy temperatures (centered per day) across height layers and (**b**) comparison of intraspecific temperature differences (centered per day and height layer) at a certain height layer for the moist and the dry period; asterisks indicate significance of intraspecific differences between the moist and dry period resulting from Tukey-Test (*p > 0.05, **p > 0.01, ***p > 0.001).

Daily interspecific temperature differences (ΔT) were found to vary significantly between species and position within the tree crown (Fig. 3, Table 2). Among them, position (layer) was found to be the key determinant (Table 2) accounting for 64% of the total variation. Across tree species the decline in median temperatures from the top to the bottom crown layer was found to be ~ 1 °C (Fig. 3a). A constant decline of temperature with height was observed for all tree species, except *Q. robur*, for which highest temperatures were observed for the middle position of the tree crown. However, as indicated by the significant interaction of species and layer in the ANOVA model, species effects on ΔT are not constant across height layers, which causes a shift in the species temperature sorting with height. While *Q. robur* and *F. excelsior* were the species with lowest temperatures at the top of the tree crown, they had highest temperatures at the bottom crown layer. An opposite height pattern was observed for *A. pseudoplatanus* and *C. betulus*, whereas *T. cordata* had intermediate crown temperatures across all height layers. Furthermore, we observed a large inter- and intraspecific variation in temperature for the top and bottom layer of the tree crown, which was found to be diminished at the middle position of the tree crown. The interspecific temperature difference, was highest (0.70 °C) at the top canopy and decreased towards 0.40 °C for the middle position and increased again to 0.51 °C for the bottom position, respectively. When further testing for shifts in interspecific temperature differences between the moist and the dry period (Fig. 3b), we found temperature differences between species to remained largely constant for the middle and bottom position of the tree crown. However, at the top of the canopy, temperature differences (ΔT) between species became less distinguished for the dry period. Additionally, we observed a significantly warmer canopy for *Q. robur* and a significantly cooler canopy for *T. cordata* when comparing ΔT for the moist and the dry period. Therefore, *T. cordata* and not the water-spending *F. excelsior* was found to provide the second highest cooling potential during the dry period.

**Table 2:** ANOVA table testing for the effect of species identity and height layer on daily differences in canopy temperatures (ΔT, temperatures mean centered per day); df = degrees of freedom, SS = sum of squares, F = F-value, *p* = *p*-value.

Predictor	df	SS	F	*p*
Species	4	3.65	13.44	< 0.001
Layer	2	344.75	2538.55	< 0.001
Species:Layer	8	63.68	117.22	< 0.001
Residuals	1842	125.08		

### Effect of environmental and tree characteristics on temperature variability between species

**Figure 4:**
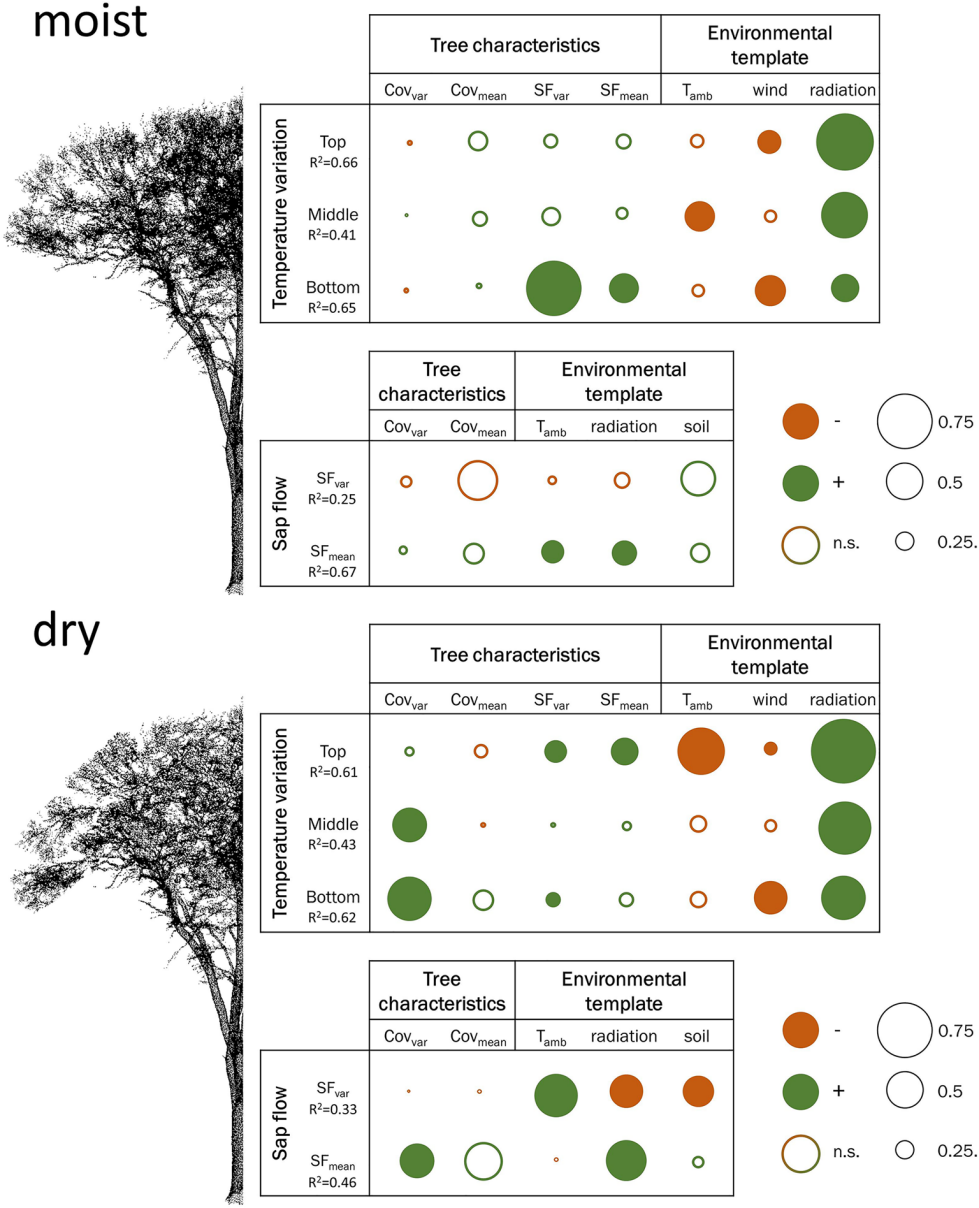
Graphical representation of estimated effect sizes of the biotic and environmental template on microclimatic variability between species (T_var_) and on the moderating transpirational performance of trees under moist and dry conditions using the SEM framework. Circle diameters corresponds to the strength of the effect; positive relationships are color-coded in green, negative relationships in red; insignificant paths are indicated by open circles, significant paths by filled circles. The effects sizes of the indirect (via sap flow) and total effects on T_var_ can be found in the supplement (Supplementary Tables S4–S6 online).

When we tested for differences in the variation of species-specific temperatures (T_var_) between the moist and the dry period (top = − 0.17, *p* = 0.022; middle = − 0.13, *p* = 0.029; bottom = − 0.11, *p* = 0.118), we found for all height layers a reduction in T_var_, which was highest for the upper canopy layers. With obtained R^2^-values ranging from 0.41 to 0.66 the proposed SEM framework was overall found to be well capable to explain the dissimilarities in canopy temperatures between tree species (T_var_) across height layers (top, middle, bottom) and hydrological periods (dry, moist). In general, we found the variance in the response variable T_var_ to be best explained for the top (moist R^2^ = 0.66; dry R^2^ = 0.61) and the bottom crown layer (moist R^2^ = 0.65, dry R^2^ = 0.62). Under moist conditions earlier in the growing season, which served as a reference in this study, the dissimilarity in canopy temperatures (T_var_), was primarily directly controlled by the environmental template (Fig. 4). In particular solar radiation was found to exert a strong positive control on T_var_, which decreased towards the sheltered bottom of the tree crown. While wind speed was negatively correlated to T_var_ at the more exposed top and the bottom positions of the tree crown, a negative relationship to ambient temperature (T_amb_) became important at the more shielded middle position of the crown. Tree characteristics only poorly explained T_var_ at the top and the middle position of the tree crown, whereas, we found a significant effect of sap flow characteristics (SF_var_, SF_mean_) on T_var_ for the bottom position of the tree crown. T_var_ was more strongly related to the interspecific variety in sap flow performances (SF_var_) than to the mean sap flow characteristics at the stand level (SF_mean_). The environmental template only moderately controlled SF_var_ (R^2^ = 0.25), with soil moisture and canopy cover at the stand level (Cov_mean_) being the most important (but insignificant) predictors. SF_mean_ on the other hand, was well explained by the environmental characteristics (R^2^ = 0.67) and significantly increased with incoming solar radiation and the interspecific variability in canopy cover.

Under dry conditions the direct impact of the environmental template on T_var_ remained largely unchanged when being compared to moist conditions, except for the high negative relationship which we observed for T_amb_ at the top canopy (Fig. 4) and the notably larger control of radiation towards lower canopy layers. But contrary to the results obtained under moist conditions, the control of canopy cover on T_var_ exceeded the importance of transpiration as soil water availability was strongly limited. T_var_ at the middle and bottom canopy layer increased with increasing variation in canopy cover (Cov_var_), while sap flow characteristics (SF_var_, SF_mean_) primarily accounted for a higher T_var_ at the top canopy. Also, the indirect control of the environmental template and characteristics of canopy cover (Cov_var_) on T_var_ via sap flow (SF_var_, SF_mean_) became apparent. SF_var_ was negatively affected by an increase in the amount of incoming solar radiation and soil moisture, but increased with increasing ambient temperature (T_amb_). Sap flow at the stand level (SF_mean_), however, increased with the amount of incoming solar radiation and with an increasing between species dissimilarity in canopy cover (Cov_var_). Detailed model results of the SEMs can be found in the supplement (Supplementary Tables S1–S3).

## Discussion

In this study we aimed at disentangling the direct and indirect effects of tree characteristics and meteorological conditions on the interspecific canopy temperature differences of five co-occurring tree species in a temperate floodplain forest being exposed to contrasting hydrological situations during the severe drought in 2018. As both, the meteorological and biophysical impact on the canopy temperature regulation change with increasing distance to the top canopy, we expected those effects to not only vary between tree species and hydrological situations, but also spatially within the tree crown. In fact, we found evidence that a large proportion of the observed canopy temperature differences is being explained by the position in the tree crown (0.64, *p* < 0.001); but also a significant tree species effect (*p* < 0.001) on canopy temperature regulation became apparent in this study. Moreover, our results suggest that the magnitude of those between species differences in canopy temperature are highest at the top and the bottom of the canopy, but the effects of tree species characteristics and the environmental template on interspecific differences in canopy temperatures vary along the height gradient. We furthermore identified a gradual shift in importance from latent heat flux to components defining the magnitude of sensible heat flux on the energy budget of trees which resulted in an equalization of tree species canopy temperatures as the drought intensified.

### Drought effects on tree characteristics and tree species differences in canopy temperature

Although broad-leaved tree species are considered to have a particular high potential to mitigate hot temperatures below the canopy^53^, we observed an average decrease in median temperatures of only ~ 1 °C (Fig. 3a) from the top towards the bottom of the canopy, which was in fact higher for *C. betulus* and *A. pseudoplatanus*, but overall markedly lower than within canopy temperature gradients for mature trees (> 5 °C; ~ 1 °C per 4 m gradient) reported in previous studies under less hot and/or dry conditions^4,44,56,57^. In line with the study of Richter et al.^9^, we found pronounced temperature ranges between the coldest and warmest tree species, which were most pronounced for the top and the bottom of the canopy (top = 0.7 °C, middle = 0.4 °C, bottom = 0.51 °C) but of overall smaller magnitude compared to the previous study.

In the following, we briefly introduce and discuss the observed drought effects on morphological and physiological tree characteristics. The local soil water availability was shown to play a pivotal role for the ability of trees to regulate the canopy temperatures, as it directly affects transpiration and thus indirectly tree health and canopy cover^3,40,58^. For all tree species investigated, canopy cover and sap flow were found to be significantly (*p* < 0.001, Table 1) decreased towards drier soil moisture conditions, suggesting that also the overall cooling potential of trees has been reduced as the drought gained severity. However, the order in which sap flow and canopy cover decreased was found to differ substantially between tree species. The recently debated^59,60^ isohydric^a^–anisohydric^b^ concept^40–42,60^, which is assumed to reflect the stomatal regulation under water limitation, well describes the observed reduction in sap flow (*Q. robur*^a^ > *A. pseuoplatanus*^a^ > *T. cordata*^a^ > *C. betulus*^b^ > *F. excelsior*^b^). Water saving tree species, showed the strongest reduction in sap flow, while for water spending tree species only a minor reduction in sap flow was observed. When analyzing sap flow reduction in the less severe drought year 2001, the study of Hölscher et al.^40^ found a similar species ranking in sap flow reduction for the overlapping set of tree species (*T.* *cordata* > *A. pseudoplatanus* > *C. betulus* > *F. excelsior*), although the species ranking of sap flow density for the initial ‘moist’ conditions differed substantially and was overall found to be lower in our study. Leaf water potential at turgor loss, or wilting, is classically recognized as a major physiological determinant of plant water stress response which can lead to a reduced canopy cover^61^. Thus, strategies of stomatal control were likewise relatable to the species ranking of decrease in canopy cover (*T.* *cordata*^a^ > *A.* *pseudoplatanus*^a^ > *C. betulus*^b^ > *Q. robur*^a^ > *F. excelsior*^b^). Due to hydraulic vulnerability of water saving tree species, drought induced premature leaf senescence occurs as a protective measure in these species, while the osmotic adjustment of mesophyll turgor at high stomatal conductance of water spending trees results in a larger plasticity in the water potential at turgor loss point^62^. The only exception was *Q.* *robur,* for which we found, despite the high relative reduction in sap flow, only a moderate reduction in canopy cover. This might be partially explained by the more xeromorphic leaf architecture of oaks being associated with a higher resistance to leaf water loss^63^.

Although the reduction of canopy cover and sap flow in reaction to drought point towards an overall reduced cooling potential of tree species, the species rank order with respect to the intraspecific temperature differences (Fig. 3b) remained, however, largely constant across height layers when comparing the two contrasting hydrological situations. Also, previous studies found the species rank order in temperature to be well preserved when analyzing time series of close-range thermal imagery data for varying environmental situations (wind, radiation, ambient temperature, atmospheric demand) under non-drought conditions for the top canopy^45^. However, contrary to the results of Leuzinger and Körner^45^, we observed a significant change in species rank order of canopy air temperatures for the uppermost canopy, when comparing dry and moist soil moisture conditions (Fig. 3b). The capability to provide to provide canopy cooling (differences ΔT between “dry and “moist”) of *Q. robur*, the species with the strongest reduction in sap flow rate, decreased towards drier soil moisture conditions. For *T.* *cordata* on the other hand, we found a significant increase in the cooling capability, regardless of the highest reduction in canopy cover and an intermediate reduction in sap flow. Hirons and Thomas^64^ described an inversion of leaves to reveal the characteristic bright reflective underside as a typical drought response for *Tilia tomentosa* moench. We observed the same phenomenon for *T. cordata* during our field campaigns, which might have resulted in a higher reflection of the solar radiation and lower leaf temperatures and may thus explain the increase in cooling potential. Changes in canopy reflectivity related to changes in leaf water status and tree phenology^65^, were, however, not considered in our study when investigation drought induces changes in the cooling potential of trees.

### Effects of tree species characteristics and the environmental template

Previous studies have shown that characteristics of tree species, such as tree morphological and physiological characteristics including transpiration rates, canopy size, and canopy density, as well as features of tree leaves, can influence the cooling potential of trees^12,33,45,66^. We expect the largest between species difference in canopy temperatures to occur at intermediate drought and heat levels, where differences in the water use strategies of trees are most distinct. However, there is little agreement in the scientific literature to what extent droughts result in an equalization of tree species temperatures^9,52,53,67^. Under severe drought conditions, our results clearly point towards a decrease in T_var_ across all height layers as trees became more stressed by limited soil moisture availability and high atmospheric demand and thermal load. The magnitude of those interspecific temperature differences, especially for the top canopy, which largely contributes to the signal captured by thermal imagery, was shown to depend strongly on characteristics of the environmental template and the tree species composition and their water use strategies (see Fig. 6). Nevertheless, the relationships between environmental and biophysical characteristics are largely missing in the previous literature. The SEM analyses revealed complex interactions between environmental and biotic drivers on differences in the cooling potential of trees (T_var_), which were found to be additionally modulated by the level of drought. Although the influence of the environmental template (T_amb_, wind, radiation) on regulating T_var_ were found to be largely constant among the two contrasting hydrological situations, the magnitudes of these effects were larger for the dry period (with the exception of wind). While in general, the importance of T_amb_ and radiation was found to be reduced towards the lower canopy layer, which is more decoupled from the atmosphere, this pattern was, however, not observed for wind. Landsberg and James^68^ have demonstrated that the reduction in wind speed is related to the canopy density, which is highest at the middle position of the tree crown. Therefore, tree species effects on microclimate regulation decreased strongest with increasing wind speed at the top and the bottom of the canopy supposedly caused by horizontal mixing effects of stable air layers due to increases in air mass transport^68,69^. On the contrary, the mixing of air layers—due to vertical turbulences and convections caused by the temperature gradient between the sunlit top and the shaded bottom canopy—is highest in the middle region of the tree crown^69,70^, which also led to a reduction in T_var_ and likely obscured the detectability of tree species effects on cooling mechanism for this canopy region. This phenomenon was also observed in a previous study by Richter et al.^9^. Variations in the warming of the air layer enclosed in the canopy at daytime and differences in heat storage capacities and conductivities of leaves and woody material^71,72^, cause a frequent increase in nocturnal between-species differences in air temperature^9^. The observed negative direct relationship between T_var_ and T_amb_ in the upper canopy regions during midday might, however, most likely resulted from vertical thermal convection resulting, in turn, from steep vertical temperature gradients at high ambient temperatures, which further increased with decreasing canopy cover towards the dry period. Among the environmental drivers, the direct effect of radiation on T_var_ was overall highest. As the amount of absorbed shortwave radiation determines canopy surface temperatures and thereby the magnitude of the sensible heat flux, the increases in air temperature are thus strongest at the sunlit top canopy^19,73^. The notably higher importance of radiation towards lower canopy layers during the ‘dry’ period likely results from the higher transmittance favored by the loss in canopy cover. However, the effect of radiation on canopy temperatures is twofold, as a healthy tree uses the absorbed radiation energy also for transpiration, which increases the latent heat at the expense of the sensible heat flux^74^. In line with Oogathoo et al.^75^, we identified radiation and atmospheric demand (T_amb_ being indicative for VPD) as the major driver of mean sap flow rates (SF_mean_). Due to a stronger stomatal regulation of the water saving tree species during the period of intensified drought, SF_mean_ was not longer found to be controlled by atmospheric demand (T_amb_) for the dry period. Although between species differences in sap flow (SF_var_) remained largely unexplained by the environmental template under ‘moist’ conditions, the increase in SF_var_ illustrates that species-specific water use strategies became increasingly visible when T_amb_ (and VPD) was high and soil water availability further decreased under ‘dry’ conditions. The combination of the positive effect of radiation on SF_mean_ and the negative effect of radiation on interspecific differences in sap flow characteristics (SF_var_) points towards a simultaneous upregulation of transpiration across tree species to allow leaf cooling at high irradiation.

As the drought affected cooling mechanisms of water saving and water spending tree differently, differences in tree characteristics were of increased importance to explainvariations in the cooling potential under ‘dry’ conditions (Fig. 4). During the moist period, where we still observed high sap flow rates across all tree species, the relationship between sap flow characteristics and T_var_ was only weak and insignificant for the upper canopy layers, but was among the dominant drivers for the lowest canopy layer. This might be explained by the high midday temperatures which could have caused stomatal regulation of the drought-sensitive water saving tree species to occur at top canopy to prevent leaf water loss^19^. However, it cannot be excluded that descending cold air masses origination from high transpiration rates at the top canopy could also have caused the relationship between T_var_ and sap flow characteristics to be detected at the lower canopy level. Despite the higher atmospheric demand and the increased need to provide sufficient leaf cooling at higher ambient temperatures during the ‘dry’ period, we found a gradual shift in importance from latent heat flux (sap flow) to components defining the magnitude of sensible heat flux (cover) on the energy budget of trees. Although, differences in the water use strategies of trees on temperature regulation became visible at the top canopy when soil moisture was limited during the dry period, differences in the latent heat flux were insufficient to compensate for components of the sensible heat flux on the energy budget of trees, which resulted in an equalization of canopy temperatures. Differences in water use strategies were, however, related to differences in canopy cover which was found to be of overall high importance for temperature regulation at the lower canopy layers. These findings suggest that under extreme drought conditions, the cooling potential of trees underneath the canopy is overall lower and largely driven by canopy cover and thus shading (see also Fig. S4 in the supplement), as it would be expected for arid environments, but not for humid environments as a floodplain forest^11^.

### Consequences for the biodiversity of forest biota

Forest biota have been shown to be sensitive towards the magnitude and the variability of forest temperatures^5,76,77^. Under changing climate, the variation of microclimatic conditions within the same habitat provides important thermal microrefugia, allowing species to evade temperature extremes^2,4,78^. Vertical and horizontal gradients of microclimatic conditions within forests are considered to shape the vertical stratification of species communities but also to impact their spatial and temporal composition^5,78–80^. Especially the abundance and diversity of smaller, short-lived and less mobile organisms is affected by the small-scale variations in micro-environmental conditions^78^. As we found evidence that the overall cooling potential of forests but also the horizontal temperature variability between tree species is reduced during severe drought conditions, this reduces the diversity of distinct microclimatic situations available within the forest canopy and might thus hamper the ability of forests to mitigate the climate change effects on forest biota. The reduced canopy cover during drought conditions furthermore decreases sheltering against incoming solar radiation, which is predicted to further increase as climate change proceeds^81^. This can result in warming of canopies beyond the thermal limits of arboricol organism such as e.g. lichens, mosses and insects^82–85^, but also enhanced the magnitude of shortwave UV radiation that can elicit stress responses and it is damaging or lethal to organisms^86,87^.

## Methods

### Study area and tree species selection

The study was conducted at the Leipzig Canopy Crane (LCC) research facility, which is located in the northwestern part of the floodplain forest of Leipzig (Saxony, Germany). The prevailing climate is continental with an average annual temperature of 8.4 °C and the mean annual precipitation is 516 mm^88^. However, during the vegetation period (April–September) in the drought year 2018 the temperature in Saxony was 3.4 °C warmer than the longtime average and precipitation was reduced by 43%^89^. The dominant soil type in the study area can be characterized as Vega, the formation of which is closely linked to upstream activities of historic human settlements and deforestation which increased soil eroding processes and led to the accumulation of a clay layer during flood events with a thickness between 1 and 4 m^90^. Leipzig´s floodplain forest, which intersects the city in northwest–southeast direction, is one of the largest floodplain forests in Central Europe^90^ European ash (*Fraxinus excelsior* L.), English oak (*Quercus robur* L.), Sycamore maple (*Acer pseudoplatanus* L.), European hornbeam (*Carpinus betulus* L.) and Small-leaved lime (*Tilia cordata* Mill.) are the dominating tree species in decreasing order according to their abundances^65^. The LCC site, which is located within a nature reserve of Leipzig’s floodplain forest, covers an overall area of 1.65 ha comprising approximately 800 trees from 17 species with diameter at breast height (DBH) > 5 cm^91^. From those, thirty individuals from five tree species (*Q.* *robur, F. excelsior, T. cordata, C. betulus* and *A.* *pseudoplatanus*) being dominant to the floodplain forest were selected for our study. For the selection of representatives of a tree species only mature individuals classified as predominant to co-dominant were considered. Tree height was measured using a Vertex system (Haglöf Sweden AB, Sweden). The DBH was measured in 2015 using a diameter measurement tape at a height of 1.3 m (Table 3). To furthermore avoid spatial clustering of tree species within our study site, tree individuals were chosen to be distant from each other and randomly distributed. A detailed map of the tree species selected can be found in the supplement (Supplementary Fig. S1 online).

**Table 3:** Morphological characteristics per species, standard deviation is given in brackets.

Species	No. Individuals	Height in m	DBH in cm
*A. pseudoplatanus*	7	29.11 (1.92)	59.37 (7.65)
*C. betulus*	5	27.47 (2.91)	54.82 (4.21)
*F. excelsior*	7	32.24 (1.19)	70.26 (17.30)
*Q. robur*	4	30.00 (2.26)	91.75 (34.49)
*T. cordata*	7	30.29 (0.72)	65.70 (13.49)

### Collection of meteorological and soil moisture data

**Figure 5:**
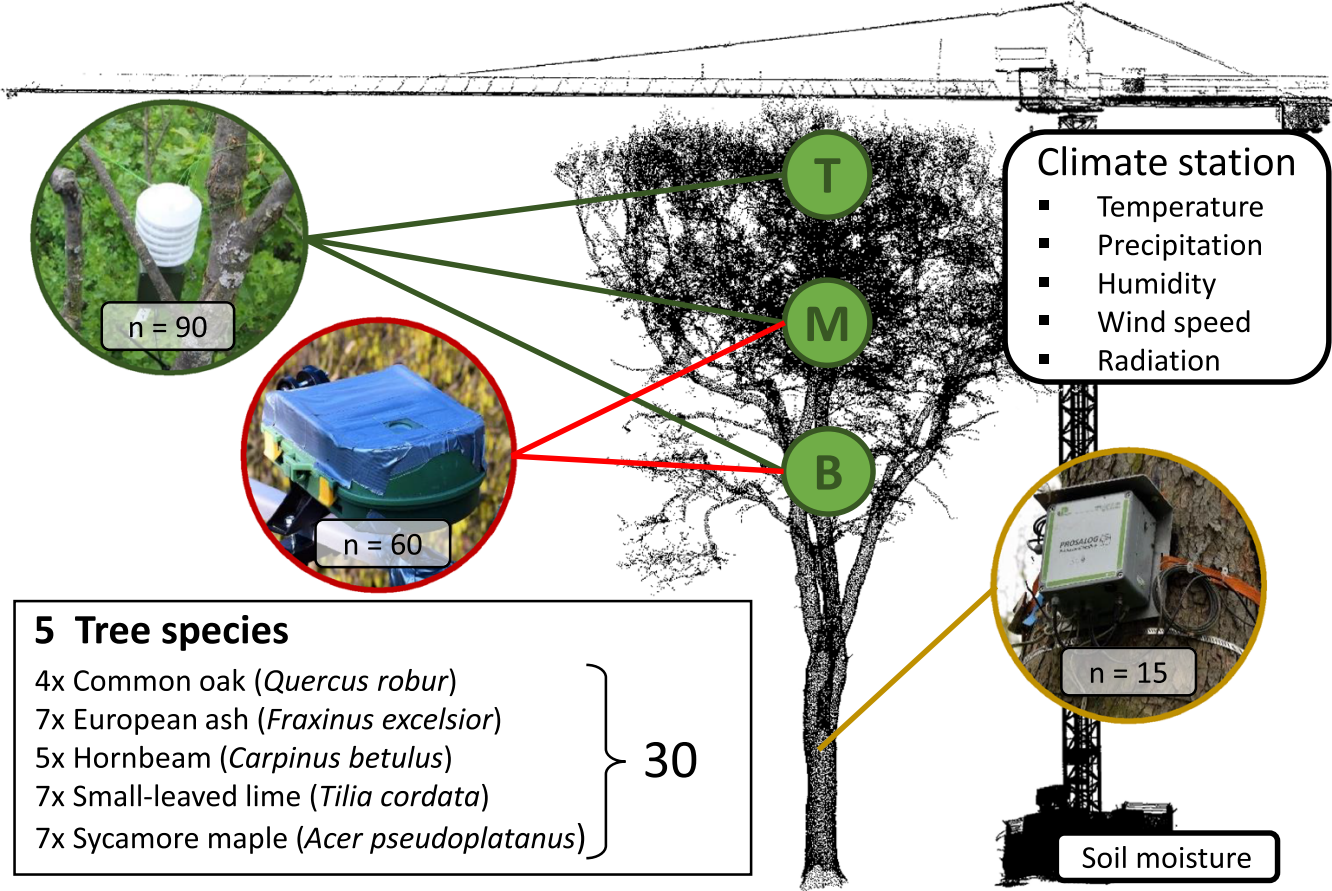
Experimental setup showing measurement locations and number of spatial replicates. Temperature sensors are highlighted in green, phenological cameras in red and the granier sap flow system in orange.

Air temperature, wind speed, precipitation, relative humidity and radiation were measured in 10-min intervals by installing a WXT520 weather sensor (Vaisala, Finland) and a SPN1 sunshine pyranometer (Delta-T Devices Ltd, England) in approximately 40 m height at the top of the canopy crane (see Fig. 5). Additionally, since Meinen et al.^92^ reported ~ 70% of tree fine root biomass to be located within the first 20 cm of the upper soil layer, we measured soil moisture (volumetric water content) at a depth of 0.1 m in 10-min intervals using a ML3 Theta-soil moisture sensor (Delta-T Devices Ltd, England). To reduce the effect of stemflow during precipitation events on soil moisture measurements, we ensured a minimum distance of 3 m to the nearest tree individual when installing the sensor. Based on the soil moisture data the investigation period was classified into two contrasting hydrological periods (see “Climatic conditions and soil moisture status in the result section”). A moist period, where soil moisture reservoirs were still sufficiently filled to supply the topsoil with water and dry period, where water supply was too limited to maintain soil moisture levels being sufficient for plant growth.

### Canopy air temperature measurements

In total, 90 temperature sensors (Scatter-100 DLTi/DLTc,UP Umweltanalytische Produkte GmbH, Ibbenbüren, Germany) were installed within the tree crowns (see Fig. 5), with three sensors per tree at three different height layers (top, middle, bottom). While top and bottom were defined as the highest and lowest layer of the foliated tree crown, middle was defined as the layer were light extinction reaches 50% (L_50_). For canopies of mature broadleaf temperate forests, L_50_ occurs at 3–4 m below top canopy^93^. The temperature sensors were equipped with radiation shield and were calibrated prior to the installation. Sensors at the bottom and middle layer of the tree crown were mounted on poles which were horizontally attached to the trunk, whereas the sensors at the very top of the tree crown were fixed at the outermost twigs using nylon cord. The sensors recorded air temperatures at intervals of 10 min, which were stored on an internal memory and later transferred to a data logger mounted at the top of the canopy crane via radio signal.

### Tree transpiration measurements

We used relative sap flow density as a proxy for tree transpiration. Sap flow density was measured with heat dissipation sensors (UP Sap Flow-System, SFS2 TypM, UP Umweltanalytische Produkte GmbH, Germany) between April and November in 2018 for three replicates per five tree species under investigation. The thermal probes were inserted 2.5 cm deep into the water-bearing sapwood at the North side of a tree at 3.5 m height above the ground to prevent vandalism. All sensors were additionally shielded against solar irradiation using reflective foil. Temperature differences (ΔT) between the unheated sensor and the heated sensor powered by a constant current of 12 V were recorded in 10-min intervals. To correct for temperature effects on differences in heat conductance, which can distort the zero-flow determination^94^, temperature dependent conversion factors were applied to convert voltage to temperature, following the instructions in the manual. Temperature differences were converted to sap flow density (Js; ml cm^−2^ min^−1^) according to Eq. (1)^95^:

1 $$Js = 0.714\left( {\left( {\frac{{{\Delta T}_{{{\text{max}}}} }}{{{\Delta T}}}} \right) - 1} \right)^{1.231}$$

whereby ΔT_max_ is the maximum temperature difference at night where sap flow is assumed to be zero and ΔT is the instant temperature difference.

### Canopy cover estimation

Canopy cover was estimated based on digital photographs, which were obtained using time laps cameras (Plant Cam–WSCA04, Wingscapes Inc, U.S.) installed facing upwards in each tree crown (see Supplementary Fig. S2 online). Each camera was adjusted to captured five images per day between 7 am and 7 pm which were stored on a SD-card. To minimize potential sources of distortions under varying weather and lightening conditions (e.g. under- and overexposure) the best suited image per camera and day was selected by visual inspection. Each image was classified into sky and canopy based on pre-defined color-classes using the digital image analyzer *WinSCANOPY Pro 2009a* (Regent Instruments Inc., Canada). This classification provides input data for the subsequent processing. Therefore, all classified images had to pass a quality check. In case of detected misclassifications, the images were digitally postprocessed and reclassified. As a proxy for canopy cover the percentage of pixels assigned to the class canopy was used. To correct for the influence of stems and branches on canopy cover estimation, we accounted for canopy cover obtained under leaf-off conditions (Cover_0_) as follows:

2 $$Cover = \frac{{Cover_{i} - Cover_{0} }}{{\left( {100 - Cover_{0} } \right)* 0.01}}$$

However, in how far the changes in canopy cover are related to changes in canopy architecture (e.g. leaf inclination) or senescence (e.g. leaf shedding) cannot be distinguished by solely monitoring canopy transparency^96^.

### Statistical analysis

As this study is explicitly focused on the period of the midday irradiation maximum, parameters representing climatic (temperature, wind speed, radiation), edaphic (soil moisture) and plant physiological characteristics (sap flow density), were averaged on a daily basis for the time period between 12:00 and 14:00 CET prior to the analysis. As it was, due to logistical constrains, not possible to measure sap flow for all single trees, we used daily midday averages of sap flow and canopy cover at the species level to characterize species-specific differences in cooling mechanisms.

#### Detecting tree species effect on temperature variations within the canopy

To test if interspecific tree temperatures differ significantly within the canopy, we performed an analysis of variance (ANOVA). As we focused on detecting interspecific differences in canopy air temperatures, we accounted for temporal differences in their magnitude via a mean-centering of temperatures per day. This resulted in temperatures (further denoted as ΔT) higher than the community mean (actual average) to be positive and temperatures lower than the community mean to be negative. The resulting model to test for the dependency of ΔT included species identity, layer and their first order interactions as predictor variables because we assumed the tree species effects on ΔT to vary within and among height layers. To additionally test for layerwise changes in canopy temperatures of species (ΔT) among contrasting hydrological situations, temperatures were mean-centered per day for each position separately and the hydrological situations was included as second order interaction to the ANOVA. Statistical comparisons at the species level were subsequently assessed using the Tukey post-hoc test.

#### Disentangling drivers of canopy temperature variability

**Figure 6:**
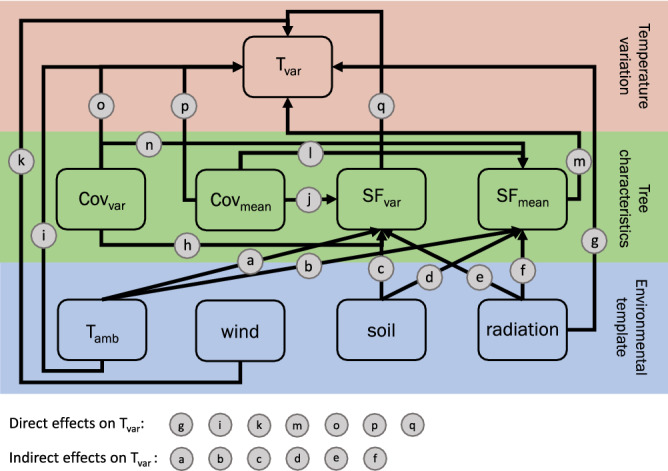
Conceptual SEM structure used to test for direct effects of tree characteristics and the environmental template on variations in canopy temperatures (T_var_) and the factors moderating transpirational performance of trees; Cov_var_ = interspecific variation in canopy cover, Cov_mean_ = mean canopy cover, SF_var_ = interspecific variation in sap flow density, SF_mean_ = mean sap flow density, T_amb_ = ambient temperature, wind = wind speed, soil = soil moisture, radiation = total solar radiation.

The temperature variation (T_var_) between tree species was analyzed using a structural equation model (SEM) which allows to analyze cascading effects of the environmental template and tree characteristics on T_var_, that would otherwise go unrecognized by any single model^97^. The environmental template included ambient air temperature (T_amb_), wind speed (wind), soil moisture (soil) and total radiation (radiation). The biotic template was characterized using the daily mean (Cov_mean_, SF_mean_) and relative standard deviation (Cov_var_, SF_var_) of the species-specific canopy cover and sap flow densities, respectively (Fig. 6).

T_var_ was defined as the daily relative standard deviation of species-specific mean midday air temperatures. Thus, a low T_var_ indicates a high similarity in species-specific air temperatures, while a high T_var_ indicates a higher variability in interspecific temperature variation. In case differences in tree species water use strategies would affect T_var_, a strong relationship of T_var_ with SF_var_ (q) can be expected, whereas a strong relationship with Cov_var_ (o) would suggest that interspecific differences in the shading potential is the dominant driver. In contrast, a strong relationship of T_var_ with Cov_mean_ (p) or SF_mean_ (m) would point towards a regulation mechanism at the community level rather than tree species differences. The environmental template being characterized by daily midday averages of ambient temperature (T_amb_), radiation, windspeed (wind), and soil moisture (soil), was included in the analysis as it affects T_var_ both directly (g, i, k) and indirectly (a–f) via moderating the transpirational performance of the trees. While direct effects of T_amb_ (i) and radiation (g) on T_var_ would point towards interspecific differences in canopy warming due to long wave radiation, indirect effects would point towards stomatal control. As the effect of wind speed on SF_var_ and SF_mean_ was neglectable and insignificant for all models tested, this relationship was not considered in the final model. This resulted in the following submodels:

3 $$SF_{{\text{var}}} \sim a_{0} + a_{e} radiation + a_{c} soil + a_{a} T_{amb} + a_{h} Cov_{{\text{var}}} + a_{j} Cov_{mean}$$

4 $$SF_{mean} \sim a_{0} + a_{f} radiation + a_{d} soil + a_{b} T_{amb} + a_{n} Cov_{{\text{var}}} + a_{l} Cov_{mean}$$

5 $$T_{{\text{var}}} \sim a_{0} + a_{q} SF_{{\text{var}}} + a_{o} Cov_{{\text{var}}} + a_{m} SF_{mean} + a_{p} Cov_{mean} + a_{g} radiation + a_{i} wind + a_{k} T_{amb}$$

In order to account for the non-independence of errors and to achieve unbiased estimates of the significance of the predictors, we used generalized least squares (gls) models implemented in the R package *nlme* ver. 3.1-155^98^ to account for a temporal autocorrelation structure of order 1 (corCAR1) that included date as covariates (as suggested by Dornelas^99^). As we expected these relationships to vary spatially as well as with the level of drought, the analysis was conducted for each combination of height layer (top, middle and bottom) and hydrological situation (dry, moist) separately. Each SEM model was evaluated using Fisher’s C statistic that can be compared with a χ^2^-distribution. If the resulting *P* value is > 0.05, then the hypothesized causal network can be considered to be consistent with the data^100^. A test of directed separations^100^ was performed on the model to test for missing possible paths, which upon inclusion would lead to an improvement of the model. We implemented SEMs using the ‘*piecewiseSEM*’ package ver. 2.1.2^97^. All statistical analyses were conducted in R ver. 4.0.3^101^.

## Supplementary Information

Supplementary Information.

## Data Availability

All data used in this article is available at https://doi.pangaea.de/10.1594/PANGAEA.944139.
