# Supplementary material for: Tree species matter for forest microclimate regulation during the drought year 2018: disentangling environmental drivers and biotic drivers
Source: Sci Rep. 2022 Oct 20;12:17559. doi: 10.1038/s41598-022-22582-6 (PMC9584904; doi:10.1038/s41598-022-22582-6)
Supplement: Supplementary file 1 — Supplementary Information. [file 41598_2022_22582_MOESM1_ESM.pdf]

Supplement to:

Tree species matter for forest microclimate regulation during the drought year 2018: Disentangling environmental drivers and biotic drivers

Ronny Richter[1,2,3], Helen Ballasus[4], Rolf A. Engelmann[1,2], Christoph Zielhofer[4], Anvar Sanaei[1], Christian Wirth[1,2,5]

[1] Systematic Botany and Functional Biodiversity, Institute for Biology, Leipzig University, Johannisallee 21, 04103 Leipzig, Germany
[2] German Centre for Integrative Biodiversity Research (iDiv) Halle-Jena-Leipzig, Puschstraße 4, 04103 Leipzig, Germany
[3] Geoinformatics and Remote Sensing, Institute for Geography, Leipzig University, Johannisallee 19a, 04103 Leipzig, Germany
[4] Chair of Physical Geography, Institute for Geography, Leipzig University, Johannisallee 19a, 04103 Leipzig, Germany
[5] Max-Planck Institute for Biogeochemistry, 07745 Jena, Germany

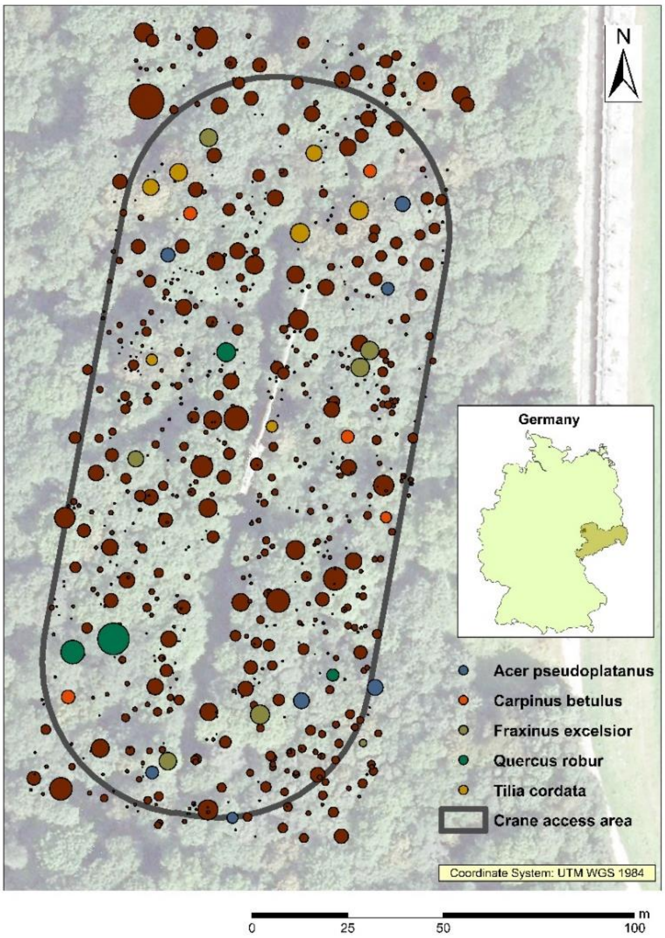

Figure S1. Leipzig canopy crane facility (LCC) and locations of the tree individuals selected for our study; species identity is color-coded; tree individuals not being selected for our study appear in brown.

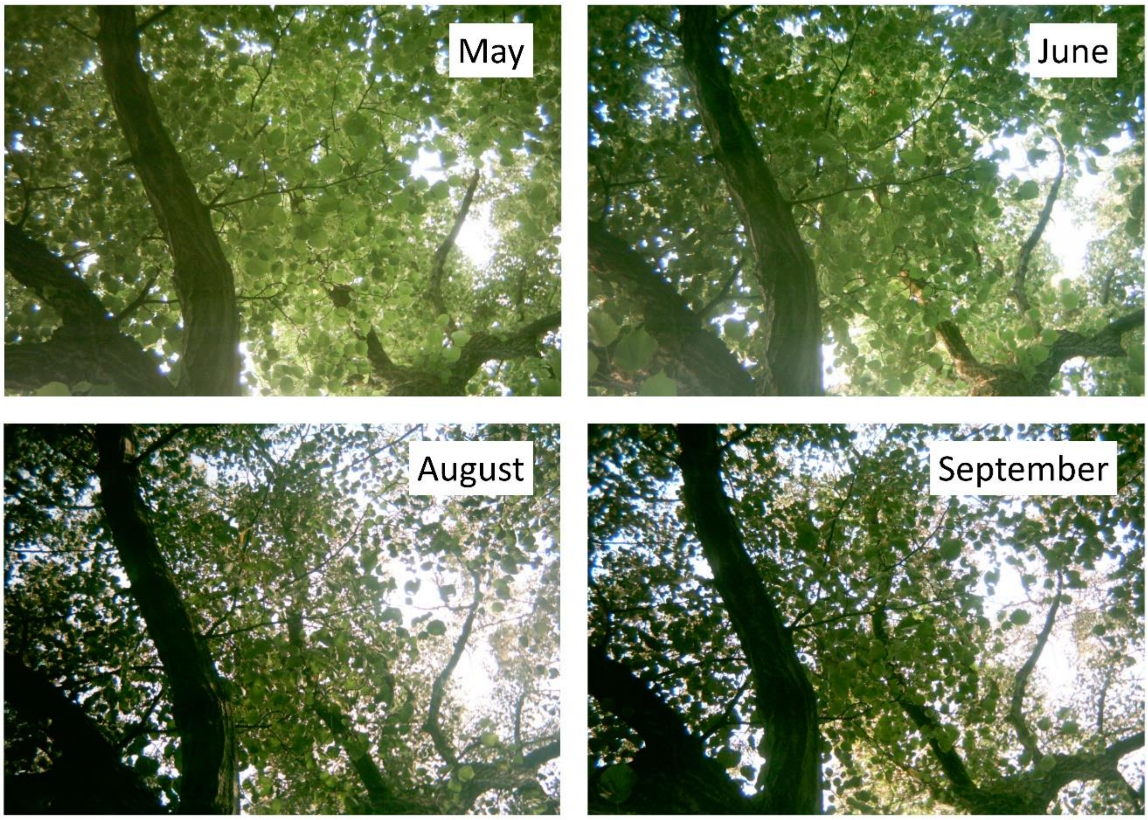

Figure S2. Images obtained in *C. betulus* used to predict canopy cover. A clear trend towards la lower canopy cover becomes visible when comparing images from the moist period in Mai / June to the dry period in August / September.

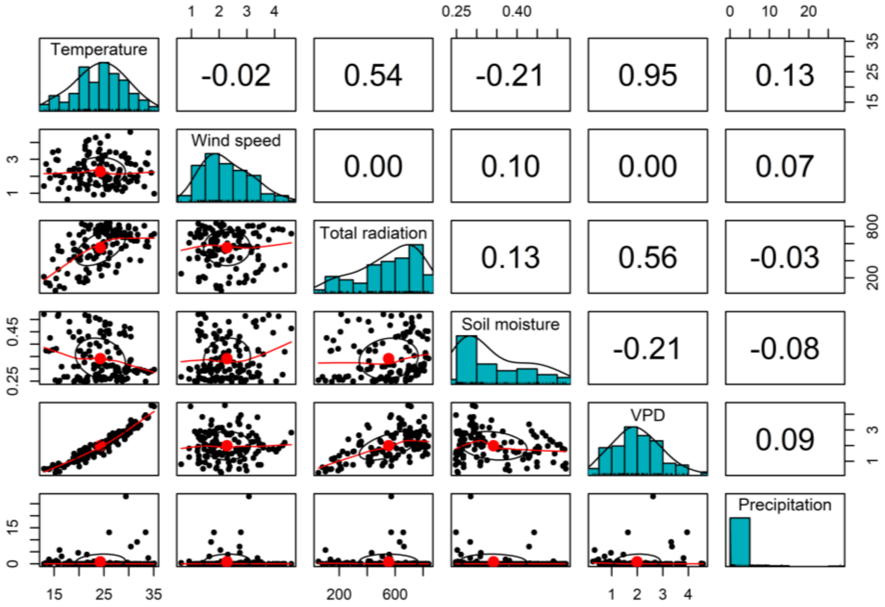

Figure S3. Bivariate relationships (Pearson correlation) between environmental characteristics measured during our study period (May 15th to September 15th in 2018).

Table S4. SEM results testing for the effects of the biotic and environmental template on between species variability in canopy temperatures (T_{var}) at the top of the canopy under dry and moist conditions; Std. Estimate = standardized coefficient, df = degrees of freedom, Crit. = critical value ratio, p – value = p – value; significant relationships are highlighted in bold.

		**moist**				**dry**			
**Response**	**Predictor**	**Std. Estimate**	**df**	**Crit.**	**p - value**	**Std. Estimate**	**df**	**Crit.**	**p - value**
SF_{var}	radiation	-0.201 (0.143)	61	-1.41	0.1638	-0.458 (0.153)	62	-3.00	**0.0040**
SF_{var}	soil	0.462 (0.256)	61	1.81	0.0764	-0.427 (0.175)	62	-2.45	**0.0176**
SF_{var}	T_{amb}	-0.103 (0.156)	61	-0.66	0.5089	0.597 (0.166)	62	3.60	< **0.001**
SF_{var}	Cov_{var}	-0.137 (0.121)	61	-1.13	0.2644	-0.027 (0.234)	62	-0.12	0.9087
SF_{var}	Cov_{mean}	-0.527 (0.270)	61	-1.95	0.0560	-0.047 (0.283)	62	-0.17	0.8693
SF_{mean}	radiation	0.347 (0.095)	61	3.65	< **0.001**	0.558 (0.137)	62	4.08	< **0.001**
SF_{mean}	soil	0.250 (0.172)	61	1.45	0.1517	0.143 (0.156)	62	0.92	0.3624
SF_{mean}	T_{amb}	0.316 (0.105)	61	3.02	**0.0038**	-0.051 (0.148)	62	-0.34	0.7317
SF_{mean}	Cov_{var}	0.097 (0.082)	61	1.19	0.2382	0.480 (0.209)	62	2.29	**0.0258**
SF_{mean}	Cov_{mean}	0.267 (0.181)	61	1.48	0.1450	0.500 (0.254)	62	1.97	0.0538
~~ SF_{mean}	SF_{var}	-0.654 (/)	61	-6.58	< **0.001**	-0.428 (/)	62	-3.64	< **0.001**
T_{var}	SF_{var}	0.183 (0.105)	61	1.74	0.0876	0.314 (0.110)	62	2.84	**0.0063**
T_{var}	Cov_{var}	-0.096 (0.097)	61	-0.99	0.3273	0.109 (0.194)	62	0.58	0.5761
T_{var}	SF_{mean}	0.191 (0.160)	61	1.19	0.2391	0.375 (0.130)	62	2.89	**0.0055**
T_{var}	Cov_{mean}	-0.246 (0.135)	61	-1.82	0.0741	-0.166 (0.211)	62	-0.79	0.4356
T_{var}	radiation	0.787 (0.102)	61	7.69	< **0.001**	0.886 (0.137)	62	6.48	< **0.001**
T_{var}	wind	-0.328 (0.074)	61	-4.43	< **0.001**	-0.187 (0.090)	62	-2.08	**0.0423**
T_{var}	T_{amb}	-0.171 (0.121)	61	-1.41	0.1644	-0.651 (0.143)	62	-4.54	< **0.001**
~~T_{var}	soil	-0.096 (/)	61	-0.73	0.2329	0.030 (/)	62	0.23	0.4079
		Fisher's C = 0.748, p - value = 0.945				Fisher's C = 2.833, p - value = 0.586			
Test of direct separation									
Independence claim		Criterion value		p - value		Criterion value		p - value	
SF_{var} ~ wind		-0.0332		0.9737		0.0844		0.9331	
SF_{mean} ~ wind		-0.3783		0.7067		-1.1382		0.2600	

Table S5. SEM results testing for the effects of the biotic and environmental template on between species variability in canopy temperatures (T_{var}) at the middle of the canopy under dry and moist conditions; Std. Estimate = standardized coefficient, df = degrees of freedom, Crit. = critical value ratio, p – value = p – value; significant relationships are highlighted in bold.

		moist				dry			
**Response**	**Predictor**	**Std. Estimate**	**df**	**Crit.**	**p - value**	**Std. Estimate**	**df**	**Crit.**	**p - value**
SF_{var}	radiation	-0.201 (0.143)	61	-1.41	0.1638	-0.458 (0.153)	62	-3.00	**0.0040**
SF_{var}	soil	0.462 (0.256)	61	1.81	0.0764	-0.427 (0.175)	62	-2.45	**0.0176**
SF_{var}	T_{amb}	-0.103 (0.156)	61	-0.66	0.5089	0.597 (0.166)	62	3.60	< **0.001**
SF_{var}	Cov_{var}	-0.137 (0.121)	61	-1.13	0.2644	-0.027 (0.234)	62	-0.12	0.9087
SF_{var}	Cov_{mean}	-0.527 (0.270)	61	-1.95	0.0560	-0.047 (0.283)	62	-0.17	0.8693
SF_{mean}	radiation	0.347 (0.095)	61	3.65	< **0.001**	0.558 (0.137)	62	4.08	< **0.001**
SF_{mean}	soil	0.250 (0.172)	61	1.45	0.1517	0.143 (0.156)	62	0.92	0.3624
SF_{mean}	T_{amb}	0.316 (0.105)	61	3.02	**0.0038**	-0.051 (0.148)	62	-0.34	0.7317
SF_{mean}	Cov_{var}	0.097 (0.082)	61	1.19	0.2382	0.480 (0.209)	62	2.29	**0.0258**
SF_{mean}	Cov_{mean}	0.267 (0.181)	61	1.48	0.1450	0.500 (0.254)	62	1.97	0.0538
~~ SF_{mean}	SF_{var}	-0.654 (/)	61	-6.58	< **0.001**	-0.428 (/)	62	-3.64	< **0.001**
T_{var}	SF_{var}	0.240 (0.145)	61	1.66	0.1023	0.048 (0.134)	62	0.361	0.7196
T_{var}	Cov_{var}	0.019 (0.121)	61	0.16	0.8747	0.482 (0.235)	62	2.05	**0.0457**
T_{var}	SF_{mean}	0.150 (0.220)	61	0.68	0.4986	0.099 (0.157)	62	0.63	0.5327
T_{var}	Cov_{mean}	0.194 (0.165)	61	1.17	0.2454	-0.050 (0.256)	62	-0.20	0.8449
T_{var}	radiation	0.643 (0.140)	61	4.60	< **0.001**	0.733 (0.166)	62	4.43	< **0.001**
T_{var}	wind	-0.152 (0.104)	61	-1.46	0.1496	-0.150 (0.111)	62	-1.35	0.1817
T_{var}	T_{amb}	-0.422 (0.149)	61	-2.83	**0.0066**	-0.210 (0.176)	62	-1.19	0.2386
~~T_{var}	soil	-0.013 (/)	61	-0.10	0.4607	-0.194 (/)	62	-1.52	0.0676
		Fisher's C = 0.748, p - value = 0.945				Fisher's C = 2.833, p - value = 0.586			

Test of direct separation				
Independence claim	Criterion value	p - value	Criterion value	p - value
SF_{var} ~ wind	-0.0332	0.9737	0.0844	0.9331
SF_{mean} ~ wind	-0.3783	0.7067	-1.1382	0.2600

Table S6. SEM results testing for the effects of the biotic and environmental template on between species variability in canopy temperatures (T_{var}) at the bottom of the canopy under dry and moist conditions; Std. Estimate = standardized coefficient, df = degrees of freedom, Crit. = critical value ratio, p – value = p – value; significant relationships are highlighted in bold.

		**moist**				**dry**			
**Response**	**Predictor**	**Std. Estimate**	**df**	**Crit.**	**p - value**	**Std. Estimate**	**df**	**Crit.**	**p - value**
SF_{var}	radiation	-0.201 (0.143)	61	-1.41	0.1638	-0.458 (0.153)	62	-3.00	**0.0040**
SF_{var}	soil	0.462 (0.256)	61	1.81	0.0764	-0.427 (0.175)	62	-2.45	**0.0176**
SF_{var}	T_{amb}	-0.103 (0.156)	61	-0.66	0.5089	0.597 (0.166)	62	3.60	< **0.001**
SF_{var}	Cov_{var}	-0.137 (0.121)	61	-1.13	0.2644	-0.027 (0.234)	62	-0.12	0.9087
SF_{var}	Cov_{mean}	-0.527 (0.270)	61	-1.95	0.0560	-0.047 (0.283)	62	-0.17	0.8693
SF_{mean}	radiation	0.347 (0.095)	61	3.65	< **0.001**	0.558 (0.137)	62	4.08	< **0.001**
SF_{mean}	soil	0.250 (0.172)	61	1.45	0.1517	0.143 (0.156)	62	0.92	0.3624
SF_{mean}	T_{amb}	0.316 (0.105)	61	3.02	**0.0038**	-0.051 (0.148)	62	-0.34	0.7317
SF_{mean}	Cov_{var}	0.097 (0.082)	61	1.19	0.2382	0.480 (0.209)	62	2.29	**0.0258**
SF_{mean}	Cov_{mean}	0.267 (0.181)	61	1.48	0.1450	0.500 (0.254)	62	1.97	0.0538
~~ SF_{mean}	SF_{var}	-0.654 (/)	61	-6.58	< **0.001**	-0.428 (/)	62	-3.64	< **0.001**
T_{var}	SF_{var}	0.762 (0.1113)	61	6.77	< **0.001**	0.208 (0.099)	62	2.12	**0.0388**
T_{var}	Cov_{var}	-0.035 (0.090)	61	-0.39	0.6959	0.612 (0.173)	62	3.53	< **0.001**
T_{var}	SF_{mean}	0.410 (0.171)	61	2.40	**0.0201**	0.182 (0.114)	62	1.60	0.1151
T_{var}	Cov_{mean}	0.063 (0.123)	61	0.52	0.6079	0.273 (0.191)	62	1.43	0.1587
T_{var}	radiation	0.388 (0.108)	61	3.59	< **0.001**	0.610 (0.120)	62	5.07	< **0.001**
T_{var}	wind	-0.434 (0.081)	61	-5.34	< **0.001**	-0.463 (0.089)	62	-5.22	< **0.001**
T_{var}	T_{amb}	-0.160 (0.110)	61	-1.46	0.1513	-0.215 (0.139)	62	-1.55	0.1271
~~T_{var}	soil	-0.021 (/)	61	-0.16	0.4379	-0.233 (/)	62	-1.84	**0.0355**
		Fisher's C = 0.748, p - value = 0.945				Fisher's C = 2.833, p - value = 0.586			
Test of direct separation									
Independence claim		Criterion value		p - value		Criterion value		p - value	
SF_{var} ~ wind		-0.0332		0.9737		0.0844		0.9331	
SF_{mean} ~ wind		-0.3783		0.7067		-1.1382		0.2600	

Table S7. Direct, indirect and total effects for single predictors on T_{var} at the top canopy position for the moist and dry period; computed from estimated coefficients (Std. Estimate) in table S4.

	moist				dry			
**Predictor**	**Direct effect**	**Indirect effect**		**Total effect**	**Direct effect**	**Indirect effect**		**Total effect**
		SF_{mean}	SF_{var}			SF_{mean}	SF_{var}	
Cov_{mean}	-0.246	0.051	-0.096	-0.291	-0.166	0.188	-0.015	0.007
Cov_{var}	-0.096	0.019	-0.025	-0.102	0.109	0.180	-0.008	0.281
SF_{mean}	0.191	/	/	0.191	0.375	/	/	0.375
SF_{var}	0.183	/	/	0.183	0.314	/	/	0.314
radiation	0.787	0.066	-0.037	0.816	0.886	0.210	-0.144	0.952
soil	/	0.048	0.084	0.132	/	0.053	-0.134	-0.081
T_{amb}	-0.171	0.060	-0.019	-0.130	-0.651	-0.019	0.187	-0.483
wind	-0.328	/	/	-0.328	-0.187	/	/	-0.187

Table S8. Direct, indirect and total effects for single predictors on T_{var} at the middle canopy position for the moist and dry period; computed from estimated coefficients (Std. Estimate) in table S5.

	moist				dry			
**Predictor**	**Direct effect**	**Indirect effect**		**Total effect**	**Direct effect**	**Indirect effect**		**Total effect**
		SF_{mean}	SF_{var}			SF_{mean}	SF_{var}	
Cov_{mean}	0.194	0.040	-0.126	0.108	-0.050	0.049	-0.002	-0.003
Cov_{var}	0.019	0.015	0.033	0.001	0.482	0.047	-0.001	0.528
SF_{mean}	0.150	/	/	0.150	0.099	/	/	0.099
SF_{var}	0.240	/	/	0.240	0.048	/	/	0.048
radiation	0.643	0.052	-0.048	0.647	0.733	0.055	-0.022	0.766
soil	/	0.037	0.111	0.148	/	0.014	-0.021	-0.007
T_{amb}	-0.422	0.047	-0.025	-0.400	-0.210	-0.005	0.029	-0.186
wind	-0.152	/	/	-0.152	-0.150	/	/	-0.150

Table S9. Direct, indirect and total effects for single predictors on T_{var} at the bottom canopy position for the moist and dry period; computed from estimated coefficients (Std. Estimate) in table S6.

	moist				dry			
**Predictor**	**Direct effect**	**Indirect effect**		**Total effect**	**Direct effect**	**Indirect effect**		**Total effect**
		SF_{mean}	SF_{var}			SF_{mean}	SF_{var}	
Cov_{mean}	0.063	0.109	-0.401	-0.229	0.273	0.091	-0.010	0.354
Cov_{var}	-0.035	0.040	-0.104	-0.099	0.612	0.087	-0.006	0.693
SF_{mean}	0.410	/	/	0.410	0.182	/	/	0.182
SF_{var}	0.762	/	/	0.762	0.208	/	/	0.208
radiation	0.388	0.142	-0.153	0.377	0.610	0.102	-0.095	0.617
soil	/	0.103	0.352	0.455	/	0.026	-0.089	-0.063
T_{amb}	-0.160	0.129	-0.079	-0.110	-0.215	-0.009	0.124	-0.100
wind	-0.434	/	/	-0.434	-0.463	/	/	-0.463

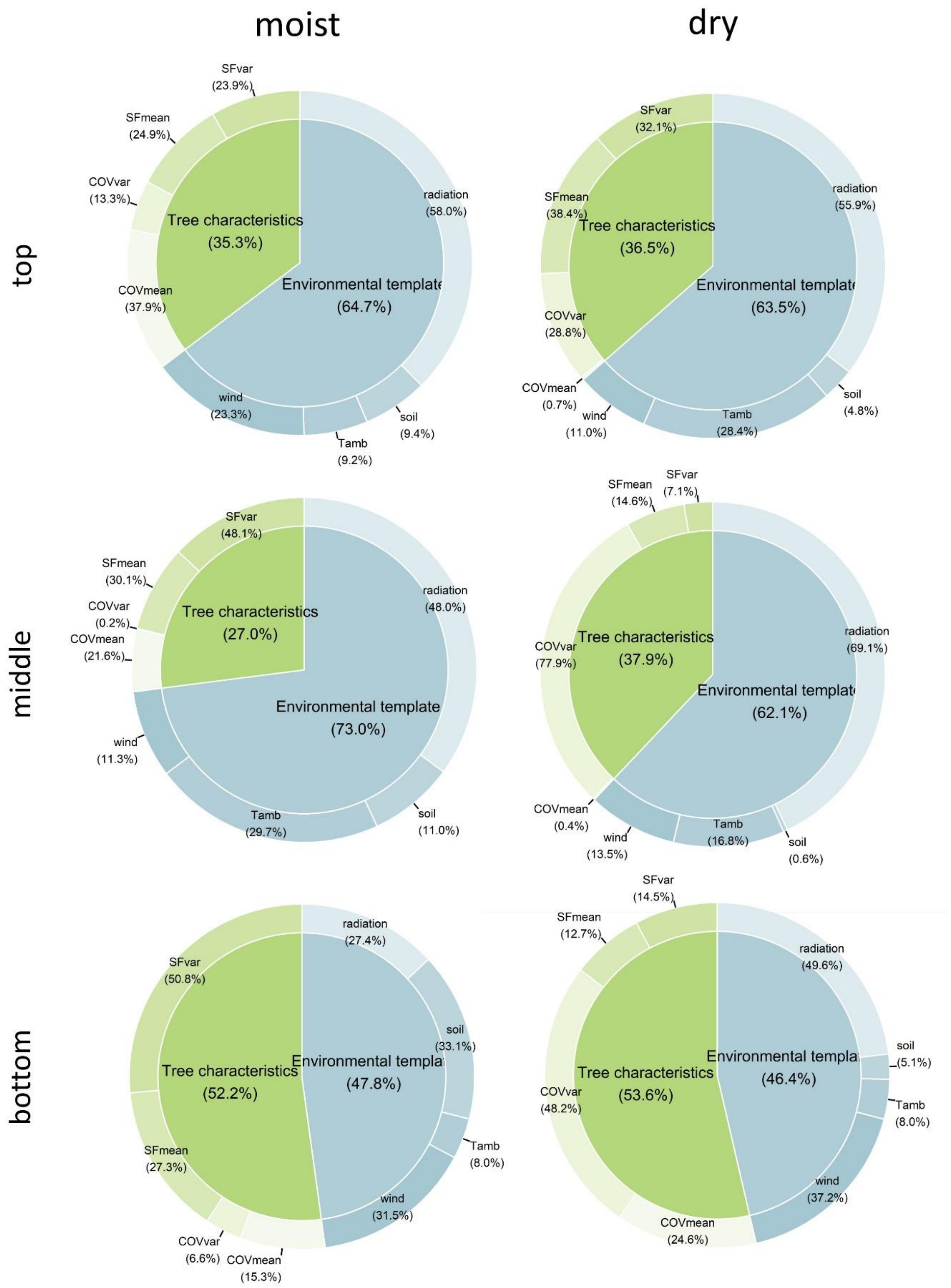

Figure S10. Pie-chart showing the relative contribution of the environmental template and tree characteristics (inner circle) and the relative contribution of single variables within both groups (outer circle) on Tvar across height layers and hydrological situations.

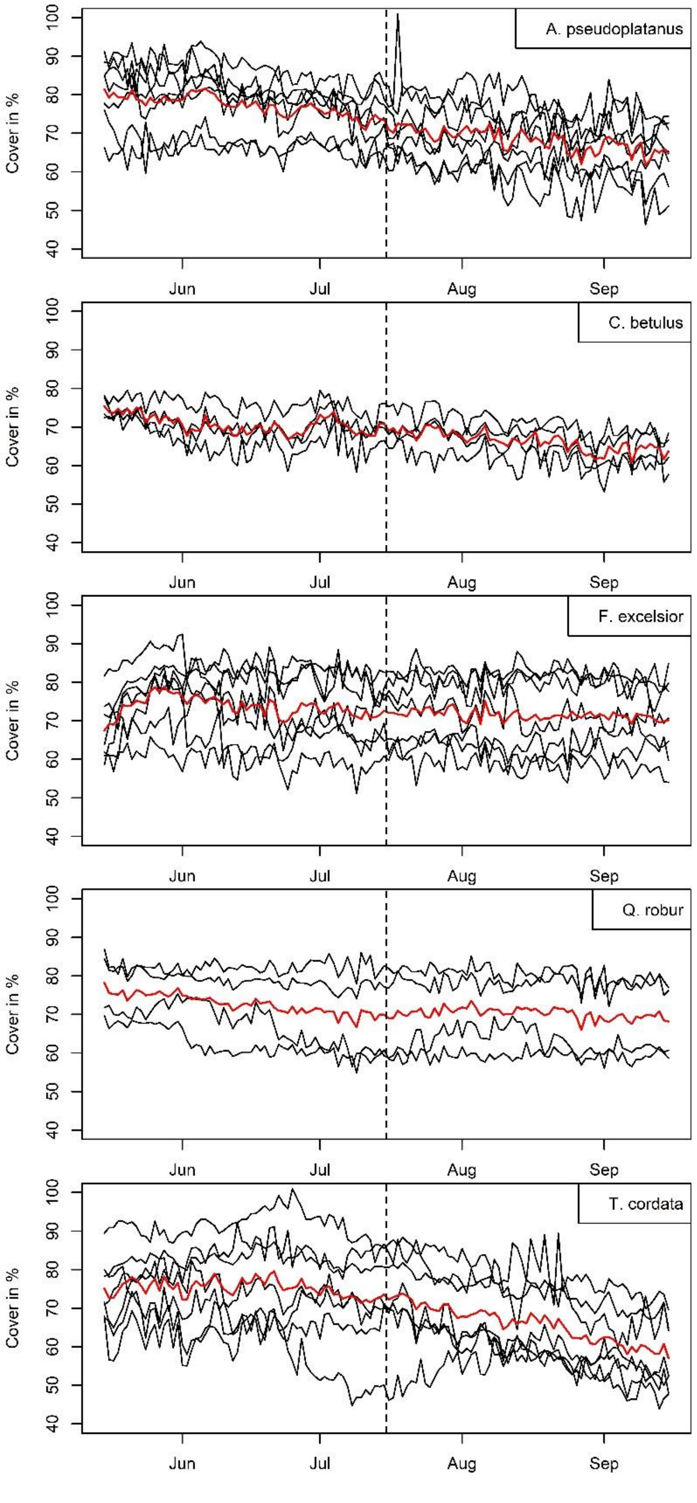

Figure S11. Predicted canopy cover per tree individual (black lines) separated by tree species; red lines indicate mean canopy cover per tree species used in the SEM-models.

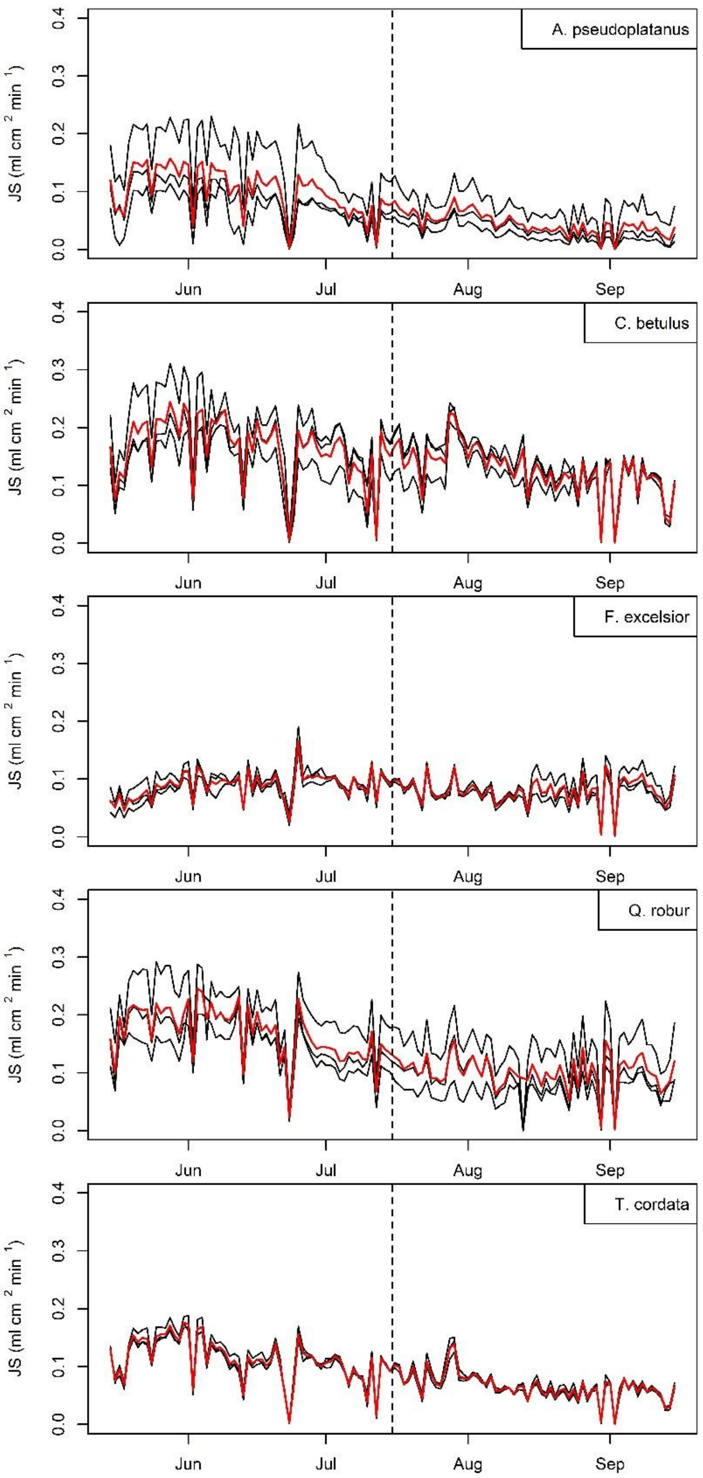

Figure S12. Sap flow density per tree individual (black lines) separated by tree species; red lines indicate mean sap flow density per tree species used in the SEM-models.

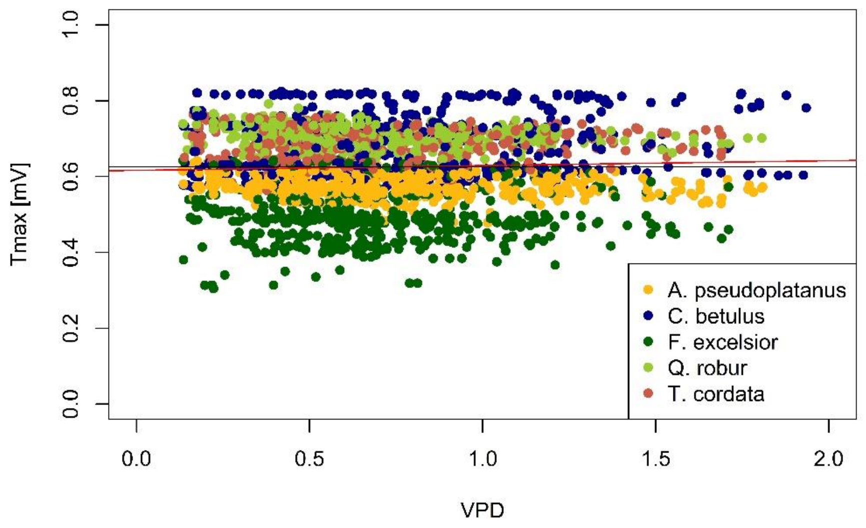

Figure S13. Daily T_{max} at nighttime, where sap flow is assumed to be zero, in relation to VPD; black line indicates mean T_{max} and red line indicates the linear relationship between VPD and T_{max}; as Tmax was not found to rely on VPD the effect on night-time transpiration on T_{max} can be excluded.

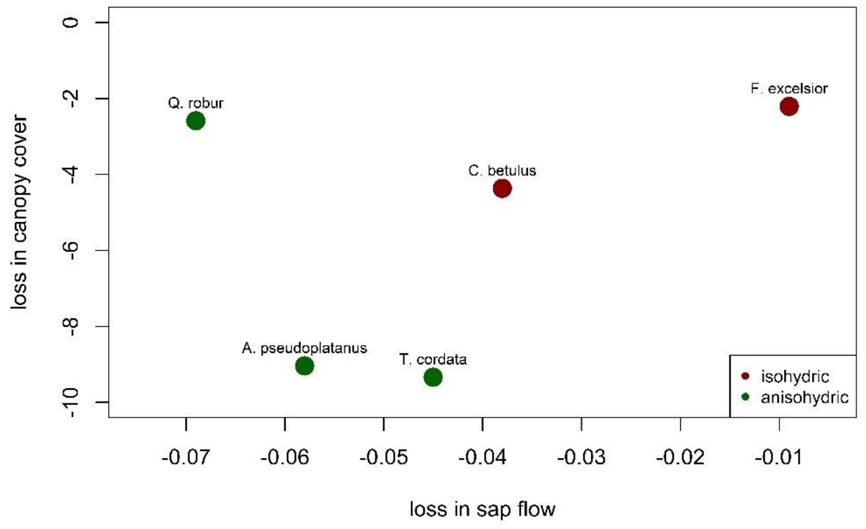

Figure S14. Relationship between loss in canopy cover and loss in sap flow density; Spearman correlation is 0.3 for all species and increases to 0.8 when excluding *Q. robur* from the analysis.

Table S15. Species-wise comparison of reduction in sap flow; red indicates less reduction; blue indicates higher reduction; reference species are presented in the rows; asterisks indicate significance level (* < 0.05, ** < 0.01, *** < 0.001).

	Ace pse	Car bet	Fra exc	Que rob	Til cor
Ace pse	x	***	***	***	***
Car bet	***	x	***	***	***
Fra exc	***	***	x	***	***
Que rob	***	***	***	x	***
Til pla	***	***	***	***	x

Table S16. Species-wise comparison of reduction in canopy cover; red indicates less reduction; blue indicates higher reduction; reference species are presented in the rows; asterisks indicate significance level (* < 0.05, ** < 0.01, *** < 0.001).

	Ace pse	Car bet	Fra exc	Que rob	Til cor
Ace pse	x	***	***	***	***
Car bet	***	x	***	***	***
Fra exc	***	***	x	*	***
Que rob	***	***	*	x	***
Til pla	*	***	***	***	x
